# Two Successful Cases of Ovariotomy

**Published:** 1863-02

**Authors:** W. H. Byford

**Affiliations:** Professor of Obstetrics, &c., Medical Department Lind University


ARTICLE IV.

### TWO SUCCESSFUL CASES OF OVARIOTOMY.

By W. H. BYFORD, M.D., Professor of Obstetrics, &c., Medical Department Lind University.

CASE I.—Mrs. H., aged 38 years, has been gradually increasing in size with ovarian dropsy, for the last four years. She first observed a small tumor in right iliac region. Before removal, it filled up the pelvis and abdomen, until the patient was as large as if at full term of pregnancy. Her suffering for several months had been very great, on account of impaction of the pelvis, and her general health was rapidly deteriorating. The tumor was multilocular; and with its contents, weighed nineteen pounds.

The operation for its extirpation was performed in presence, and with the assistance of Drs. SHUMWAY, CHEENEY, DAVIS,

BEVAN, and JONES. An incision between three and four inches in length in the linea alba, about equal distances from the umbilicus and pubis, enabled us to draw off the contents of the numerous cysts with the trochar, and extract the whole tumor with great facility and dispatch. The pedicle was transfixed by a needle carrying a large double hempen ligature, and tied in two parts firmly as I could draw the twine. The wound was closed by three silver pins, the lower of which was passed through the middle of the pedicle, so as to confine the cut edge upon a level with the skin of the abdomen. I should have mentioned, that after being tied, the pedicle was divided between the ligature and the tumor with the knife. When the tumor was first exposed, there were within view, ramifying over its surface, a number of large veins, several of those were larger than a goose-quill. The pedicle was large and fleshy, showing several large venous trunks. There were no adhesions anywhere; and the only obstacle to a ready removal of the tumor, when lessened by evacuation, was caused by a cyst that completely filled up the cavity of pelvis. It was so completely moulded into the inequalities of that cavity, that some force and address in manœuvering were requisite to lift it out. The wound was covered with a water compress, and the abdomen encircled with a broad woollen binder. The operation and dressings were finished in eighteen minutes, after the patient was completely under the influence of chloroform. Dr. SHUMWAY, whose patient she was, and to whom much credit is due, for his assiduity and skill in the after management of the case, kept full and accurate notes; they are highly interesting on several accounts, and will be read with profit by those who are studying the subject. They are subjoined:—

Operation was completed at 3 o'clock and 33 min., Oct. 29th. 4, P.M., vomited once a little water; pulse 76; complains of smarting pain at seat of wound, and severe pain in the bowels: "like the pain of colic;" ordered tinct. opii. gtts. 40. 6, P.M., much more comfortable; says that she has not a particle of pain in the right side, but complains of pain in the left hypogastric region and down the left thigh; pulse 88, full and soft. 6.30,

P.M., pain continuing, gave tinct. opii. 30 gtts. 8.30, P.M., still complains of severe pain in left hypogastrium and thigh; pulse 88; gave tinct. opii. 40. gtts. 11.30, P.M., patient easier; has slept, with a few minutes intermission now and then, since 9 P.M.; pulse 88, as before. 12, P.M., return of pain; gave tr. opii. 30 gtts. Thursday 30th, 3, A.M., used catheter at patient's request; comfortable; pulse 88. 9, A.M., pulse 90, full and soft; complains of return of pains in the left thigh; gave tinct. opii, 30 gtts. 12, M., complains of thirst, otherwise very comfortable; ordered *ice, ad libitum;* repeat tr. opii. 30 gtts. 3, P.M., symptoms unchanged; relieved bladder with catheter; tinct. opii. 30 gtts. 5.30, no change, patient comfortable. 9, P.M., patient cheerful; ordered tr. opii. continued every three hours if she is restless, or complains of pain. Friday 31st, 8.30, A.M., found patient comfortable; has slept at intervals during the night; pulse 90; skin cool; does not complain of pain, except a slight smarting at the seat of wound; cath. 8.30, P.M., finds patient feverish; pulse 100; countenance anxious; skin dry and hot; very restless and irritable; much troubled by secretion of mucus in the trachæ, with desire to cough; slight fulness of the abdomen; had taken 40 drops tinct. opii. at 4, P.M., ordered 40 drops more, as she was still unable to pass water; her bladder was relieved by use of catheter. 10, P.M., less bronchial irritation; continue tr. opii. 30 gtts. every three hours. Saturday, Nov. 1st, 8.30, A.M., more comfortable; pulse 96, softer and fuller; has slept at intervals during the night; tympanites increased; ordered em. terebinth; continues tr. opii. 12, M., considerable tympanites; complains of pain in the bowels, with desire to go to stool; upper part of the wound looking well; slight phlegmonous inflammation about the pedicle; pulse 100; continue em. terebinth and tr. opii. 6, P.M., tympanites increased; but less tenderness over the abdomen than there was last night; more cheerful; pulse 96, soft and full; gave 5 grs. calomel; continue em. terebinth and tr. opii., with b. c. soda. Sunday, Nov. 2d, 8.30, A. M., found patient cheerful, she says that "she is almost well, and meant to have had her clothes changed before the doctor came;" nurse was engaged making her toilet; ordered all operations of that kind to be suspended; pulse 100; tympanites increased, but no tenderness over the abdomen; continue em. terebinth, tinct opii. and *perfect rest.* 4, P.M., found patient much worse; was taken soon after we left in the morning with severe pain in the left hypogastrium; pulse 128, small and quick; skin hot and dry; tympanites or flatulence much increased; gave tr. opii. 40 gtts., and ordered enema of

Ol. Terebinth,..................................℥ss.

Ol. Ricini,.......................................℥i.

Yolks of 2 eggs,..............................

Gruel,.............................................Oj. M.

6, P.M., enema retained; gave another of soap-suds, which brought away a large amount of gas, but no feculent matter; pulse 120; pain subsided; abdomen tender; wound looking tolerably well; union appears firm at the upper part; considerable phlegmonous inflammation about the pedicle; ordered the em. terebinth and tr. opii. continued every three hours, and one grain sulph. quina to be added to each dose, and the abdomen covered with a poultice of flax-seed meal, after being rubbed with ol. terebinth; care being taken not to get any of the oil into the wound; gave fl. ext. rhei. and senna, ʒij. Monday, 3d Nov., 8.30, A.M., patient rested tolerably well through the night; pulse 116; abdomen very much distended; wound firmly united at the upper part, but looking unhealthy about the ligatures; cut the ligatures, and removed two of the needles; a serous discharge followed the withdrawal of the needles; ordered beef-tea and milk-punch freely; pinned a bandage tightly around the abdomen; she does not complain of pain upon pressure; repeat fl. ext. rhei. et. senna. 12, M., abdomen enormously distended; patient restless; pulse 120; gave enema, which brought away a large amount gas, which much relieved the distention of the abdomen; repeat the rhei. and senna. 6, P.M., pulse 120; distention very great; applied adhesive-straps to support the wound; complains of no pain or tenderness; bowels have not moved; ordered enema, which only brought a discharge of gas; gave 12 grs. calomel. Tuesday 4th, 8.30, A.M., bowels have not moved; pulse 116; only complains of the distension of the abdomen; ordered bot. citrate magnesia. 2, P.M., the bowels not having moved, gave enema of ol. terebinth, which produced a free movement of the bowels, with discharge of a large quantity of gas, almost entirely relieving the abdominal distention. 5.30, P.M., patient expresses herself as "almost well;" pulse 108; skin cool; ordered injection, repeated in three hours if the bowels do not move again in the meantime; continue milk-punch and beef-tea, and give tinct. opii. 30 gtts., after the bowels move again. Wednesday 5th, 9, A.M., the injection last evening produced a complete collapse of the abdominal wall; patient slept nearly all night,—is very comfortable this morning; pulse 106; no pain, no tenderness, except at seat of wound. 5, P.M., improving steadily. Thursday 6th, 8.30, A.M., pulse 100; no unpleasant symptoms; wound around the pedicle discharging freely; takes considerable nourishment. Friday 7th, same as above. Saturday 8th, removed the remaining needle which transfixed the pedicle,—ligature firm. Sunday 9th, wound looking well; patient looking and feeling very well,—appetite good.

Case. II.—I was called, Nov. 5th, 1862, to see Miss P., aged 20 years, at Eleroy, Illinois, suffering with ovarian dropsy. There had been nothing remarkable in the condition of her health, although of rather spare and fragile form, until February last, when she first observed a tumor almost the size of an orange, in the right iliac region. Her menses ceased to make their regular appearance about the same time. The tumor had grown quite rapidly; and she had suffered for several weeks severely from pressure. At the time of examination, she seemed larger than most women at full term of pregnancy. The patient was examined by, and in consultation with, Drs. L. A. Mease, B. J. Buckley, F. W. Hance, E. C. De Puy, and John Charlton, of Freeport, Dr. R. Hays, Lena, and Dr. J. A. Darling, of Eleroy. It was unanimously decided that the tumor was multilocular, and as it had grown so rapidly, and the patient had begun to suffer from its great size,—she could not long survive, if not relieved,—that extirpation was the only means of cure advisable, and that owing to the probabilities of a large portion being solid, and the existence of adhesions, the chances of success were less than ordinary. The conclusions of the consultation being submitted to the patient; with a heroic determination, that I think had much to do with her recovery, she begged us to give her what she considered the only chance of escape from a lingering and sure death. With the assistance of the above-named gentlemen, the operation was perforned in the following manner:—After anesthesia was induced by chloroform, an incision in the linea alba, midway between the umbilicus and symphisis pubis, about two inches long, exposed the tumor and evacuated several pints of peritoneal effusion. Upon introducing the finger to survey the tumor, some slight adhesions were torn through. A large trochar was next plunged into one of the presenting cysts, as no fluid flowed out of the canula, it was withdrawn. Attached to it was a thick glutinous semi-fluid, that was so tenaceous as to admit of being drawn into a string, two feet long. It was evident that the contents of these sacs could not be thus evacuated. The external incision was enlarged until it was about five inches in length. The abdominal walls pressed closely against the tumor; a free incision made into the cyst, and the contents, almost as thick and dark as tar, pressed out. The same procedure was repeated upon several sacs, until the size of the tumor was considerably decreased. Upon drawing the partially, collapsed tumor forward, and examining its sides, firm and extensive adhesions were discovered in every direction in which the examination was pushed. Much of them gave way under the fingers, by using considerable force; there were, however, firm bands of fibrin, from two to three fingers wide, two of them were far around toward the spine, which, on account of their firmness, had to be separated by the ecrasseur, The external wound was again enlarged upward, until it was about nine inches in length, and the tumor lifted out of the abdominal cavity. After passing a double hempen ligature through the centre of the pedicle, and securing it by tying each side firmly as possible, the chain of the ecrasseur was passed through it, close to the tumor, and above the ligature. Owing to the careful attention of the gentlemen present assisting, very little of the contents of the sacs, and, probably, no blood found their way into the abdominal cavity. The external wound was now closed by four pins and several silver sutures. The stump was transfixed, and retained in the wound with its surface even with the external surface, by the pin nearest the pubic extremity of the cut. I should have before stated, that the great omentum which lay on the upper part of the tumor, was adherent throughout the whole extent of contact, but these adhesions were so feeble that they gave way under pretty smart force exerted by the fingers for the purpose.

Very little blood was lost; and the patient bore this terrible operation without any appearance of shock or depression whatever. The time occupied in completing the operation and dressing, was forty-five minutes. A compress wet with water was placed over the wound, and secured by a broad flannel binder. The tumor with its contents weighed 30 pounds. After witnessing the extensive adhesions, peculiarity of contents of the tumor, &c., all present joined in expressing the opinion that recovery was hardly to be thought of as a possibility. It will be noticed, that in these two operations, although as great care as practicable,—on account of the difficulties of the case,—was observed in avoiding extravasation or effusion in the peritoneal sac, no effort by sponging or wiping among the intestines, was made to remove any substance that did escape. Such was the case also in the first operation I ever performed; and I cannot but express the conviction, that the amount of ovarian fluid should be very considerable or acrid in quantity, to justify the rough operation of sponging it out. The notes of the case, after the operation, were kept and forwarded to me by Dr. J. A. Darling, of Eleroy, and I have not altered them, believing them to be a faithful exhibit of the progress toward a cure. Although somewhat lengthy, they show but one interesting circumstance, which is, that "our patient" recovered from the effects of the operation without a single bad symptom:—

Operation finished Nov. 5th, 12.30, P.M.; pulse feeble, and 120 per minute; countenance pale; nausea and vomiting from effects of chloroform; complains of pain in back; took teaspoonful of tinct. opii. 2.30, P.M., nausea continues; less pain in back; lies on side, disposed to doze; pulse 120, good volume; natural color returning to face; respiration 40 per minute. 5 o'clock, P.M., pulse 112, full and soft; resting well; respiration 40 per minute; complains of pain in back; some thirst; took teaspoonful of tinc. opii., which was immediately rejected; 7 o'clock, P.M., pulse 104; nausea; skin cool, soft, and natural; ¼ gr. morphia, which was immediately rejected; complains of pain in back; slight desire to urinate, which was relieved by catheter; has slept some last two hours. 8 o'clock, P.M., pulse 120; more urgent nausea, otherwise comfortable; ¼ gr. morphia immediately rejected. 8.45, P.M., ¼ gr. morphia retained. 10 o'clock, P.M., ¼ gr. morphia; some restlessness. Nov. 6th, 2 o'clock, A.M., ¼ gr. morphia; dozing. 5 o'clock, has slept some; ¼ gr. morphia; pulse 125; desire to urinate, relieved by catheter. 8 o'clock, A.M., pulse 118; skin cool, soft, and natural; ¼ gr, morphia; some nausea. 10 o'clock, A.M., ⅛ gr, morphia. 11 o'clock, pulse 120; inclined to doze. 1 o'clock, P.M., skin moist; breathing good; pulse 120, and soft. 1.15, P.M., removed bandage,—wound looking healthy; no pain or tenderness; desire to urinate, relieved by catheter; pulse 108; 1 gr. opii. (Tilden's preparation;) skin soft and cool; no nourishment taken. 3 o'clock, P.M., took a little crust coffee. 3.45, P.M., 1 gr. pill opii., with crust coffee for drink; inclined to doze. 5 o'clock, P.M., pulse 120; complains of back, otherwise comfortable. 5.30, P.M., 1 gr. pill opii.; complains of occasional shooting pains in abdomen; pulse 129. 7.15, P.M., 1 gr. pill opii. rejected. 7.30, P.M., 1 gr. pill opii. retained; inclined to doze. 8.45, P.M., 1 gr. pill opii. 10 o'clock, relieved bladder by use of catheter. Nov. 7th, 6.30 A.M., have given through the night 1 gr. pill opii. every hour and a-quarter; patient has rested well; skin cool; respiration natural; has no pain, except in back.

Nov. 9th. Our patient, thus far, is exceeding our most sanguine hopes. I will continue notes as taken from my book:—

Nov. 7th, 3 o'clock, P.M., pulse 120; tongue, some dry and red at tip, with thirst; no nausea. 5 o'clock, P.M., saw patient with Dr. CHARLTON; commenced the use of ess. beef, which relishes well; removed bandage,—wound looking well; have decreased the opii. to 1 gr. every four hours; tongue dry and slightly coated. Nov. 8th, 3 o'clock, A.M., patient resting well; tongue more natural; pulse 135. 7 o'clock, A.M., pulse 130. 10 o'clock, A.M., removed bandage,—wound looking good, with very slight suppuration; pulse 130; commenced the use of Tilden's f. ext. veratrum viride, 2 min. every two hours, continuing 1 gr. opii. every four hours; patient cheerful and happy; continues use of beef ess. and crust coffee; tongue moist. Nov. 9th, 8 o'clock, A.M., have continued above treatment through the night,—patient rested well; slight sweating when sleeping; complains some of flatus; gave 3 gr. carb. soda; pulse 110, with all other symptoms favorable; have discontinued use of verat. viride. I would here state that I have emptied the bladder regularly. 5 o'clock, P.M., saw patient with Drs. BUCKLEY and CHARLTON; removed bandage,—wound looking healthy; slight suppuration,—healing mostly by first intention; pulse 120; tongue moist; skin cool. Nov. 10th, 7 o'clock, A. M., patient has rested well through the night; pulse 120; tongue slightly dry; has no pain; have given through the night opii. as usual, with verat. viride every four hours; patient feels well and cheerful,—thinks she has grounds for hope that she will recover; there is but slight distention of abdomen.

Nov 12. Our patient is prospering finely; thus far, everything looks favorable for a recovery. Her pulse, this morning, is 84; tongue moist and clean; the only disagreeable symptom is wind in the bowels. I moved them yesterday, and shall give another injection this morning. I have continued treatment with opii. and verat. viride, the same as at first, also anise-seed tea. I am giving her all the nourishment she will take, in a liquid form.

Nov. 14th. Our patient is still doing well, has no fever, nor any unpleasant symptoms. I have moved the bowels: I used simply an injection of soap-suds, with a little turpentine. She begins to eat toast and some roast potato, with a good supply of beef essence. The wound is looking well. The superior one-half is entirely healed. I have removed one pin and two sutures, shall remove one pin to-day. I have continued treatment same as formerly.

Nov. 17th. Our patient is gaining as fast as could be expected. The wound is healing gradually, the superior one-half is entirely closed, the other is suppurating some. I have removed two of the pins and the three sutures, the others I shall allow to remain for a few days. I am treating her now with opium and quinine, 1 gr. each, every four hours. Her pulse is about 110 per minute, soft; tongue clean and good; skin soft and moist; and all appearances favorable. We have strong hopes of a recovery; I am giving her plenty of nourishment.

Nov. 19th. Our patient is gaining as fast as we could hope for,—she begins to have an appetite for food. I am continuing treatment with opium and quinine; the wound is healing slowly. I have not removed the two lower needles, yet the stump looks well. I have moved her bowels with injections about every other day, they have moved once without any medicine whatever. I see nothing to hinder a favorable termination.

Nov. 22d. Dear Sir:—Your favor of 19th was duly received. I will first answer your questions:—1st. There has not been any hemorrhage whatever from the stump or wound. 2d. There has not been distention of the abdomen at any time of any account, she only complained one day of flatus.

She is now getting along as well as could be asked for: has a good appetite, feels cheerful, and says she wants to sit up. I have removed three of the pins; I thought it was best to allow the lower one to remain for a few days, yet the stump is about on a level with the abdomen. I am giving her quinine with reduced doses of opium.

February 14th. Both patients have completely recovered from the operation, and are in good health.

